# Linking Bi-Metal Distribution Patterns in Porous Carbon Nitride Fullerene to Its Catalytic Activity toward Gas Adsorption

**DOI:** 10.3390/nano11071794

**Published:** 2021-07-09

**Authors:** Parisa Nematollahi, Erik C. Neyts

**Affiliations:** Research Group Plasmant, NANO Lab Center of Excellence, Department of Chemistry, University of Antwerp, 2610 Antwerp, Belgium; Erik.Neyts@uantwerpen.be

**Keywords:** C_24_N_24_, porphyrin-like, porous fullerene, bi-metal doping, gas adsorption, nanocage

## Abstract

Immobilization of two single transition metal (TM) atoms on a substrate host opens numerous possibilities for catalyst design. If the substrate contains more than one vacancy site, the combination of TMs along with their distribution patterns becomes a design parameter potentially complementary to the substrate itself and the bi-metal composition. By means of DFT calculations, we modeled three dissimilar bi-metal atoms (Ti, Mn, and Cu) doped into the six porphyrin-like cavities of porous C_24_N_24_ fullerene, considering different bi-metal distribution patterns for each binary complex, *viz.* Ti_x_Cu_z_@C_24_N_24_, Ti_x_Mn_y_@C_24_N_24_, and Mn_y_Cu_z_@C_24_N_24_ (with x, y, z = 0–6). We elucidate whether controlling the distribution of bi-metal atoms into the C_24_N_24_ cavities can alter their catalytic activity toward CO_2_, NO_2_, H_2_, and N_2_ gas capture. Interestingly, Ti_2_Mn_4_@C_24_N_24_ and Ti_2_Cu_4_@C_24_N_24_ complexes showed the highest activity and selectively toward gas capture. Our findings provide useful information for further design of novel few-atom carbon-nitride-based catalysts.

## 1. Introduction

Porous carbon-based catalysts are widely used as sorbents and support materials in heterogeneous catalysis [1,2,3]. The archetypical example is C_60_ fullerene, which has a closed-cage structure and can be synthesized with a highly defective surface and abundant holes [4,5]. It has high thermal stability, unique mechanical properties, high electronegativity, and high electron affinity [6]. Therefore, both pure and metal-doped C_60_ show promising applications in energy conversion [7,8], fuel cells [9,10], and for biomedical applications [11].

Recently, the adsorption and conversion of gas molecules on doped C_60_ fullerene by single transition metal (TM) or heteroatoms, especially nitrogen atoms, has gained significant interest [12,13]. Upon N-doping, carbon π electrons are activated by conjugating with the lone-pair electrons from N. Thus, the C atoms neighboring N become active centers for catalytic reactions. For instance, Chen et al. [14] theoretically investigated the oxygen reduction reaction (ORR) mechanisms and catalytic abilities of pure and N-doped fullerenes of various sizes (C_20_, C_40_, C_60_, and C_180_). They found that the two pure and N-doped C_20_ and C_180_ structures are not active toward the adsorption of common intermediates produced during the ORR process. In contrast, C_39_N showed the largest decrease in reaction energy of the rate-determining step in the relative energy profile, suggesting its ORR activity is the best among all the different sizes of fullerenes. Experimentally, N-doped carbon materials are prepared using chemical vapor deposition (CVD) or reactive magnetron sputtering [15,16]. For example, Usachov et al. [17] synthesized a N-doped graphene nanosheet from *s*-triazine molecules. Recently, Zhai et al. [18] for the first time synthesized metal-free N-doped graphene films on glass through plasma-assisted hot filament CVD using N_2_ gas as dopant. They found that both the hot filament and plasma source are essential for growing N-doped graphene of high quality. By adjusting the N_2_ flow, the authors could easily modulate the N content, transmittance, and electrical properties of the graphene films.

The chemical inertness of pure fullerenes prevents their possible application for gas capture. Single metal doping (M-C_60_), like C_58_Pt and C_59_Pt [19], significantly modifies the electronic structure of C_60_, rendering it chemically active. However, one of the main challenges in the synthesis of M-C_60_ is the often-observed aggregation of metal atoms [8,20]. Recently, extensive efforts have been put into developing single-site catalysts such as pyrolyzed TM-modified porphyrin complexes (TM = Fe, Co, Mn, Cr, Ni) [21,22,23,24,25,26,27,28,29,30,31], which can be used in various applications like ORR and batteries [24,32,33,34,35,36]. The single TM atom doped in these porphyrin units was firmly fixed, preventing metal aggregation. Recently, the porphyrin-like porous C_24_N_24_ has been of great interest as truncated N-doped C_60_ nanocage. The C_24_N_24_ fullerene has six N_4_ cavities with eight s-triazine rings, which are connected via C-C bonds. Each porphyrin-like N_4_ cavity of C_24_N_24_ can host a single TM atom. Recently, TM-doped C_24_N_24_ and C_24_B_24_ fullerenes were studied for hydrogen storage [37,38,39], ORR [40], and gas conversion [41].

In this work, we investigate the catalytic activity of bi-metal atom doping in C_24_N_24_, using Ti, Mn, and Cu as metals. In particular, we here focus on the influence of the metal atom distribution in the catalytic activity of the modified structures. We carefully investigated the adsorption characteristics, electronic properties, and charge transfer properties of our novel catalysts. We found that varying the TM ratio indeed has an important effect on the properties and catalytic activities of the catalysts toward adsorption of gas species. Our calculations can provide the fundamental adsorption mechanism of such a novel material, supporting its possible exploitation to be applied as a green catalyst for gas detection.

## 2. Computational Details

All quantum chemical DFT computations are performed using the Gaussian16 package [42]. We first tested fourteen DFT functional/basis set combinations (including GGA, meta-GGA, and hybrid functionals) for geometry optimization (see Table S1 of Supplementary Materials). As can be seen in Table S1, the obtained E_b_ values calculated with the B3LYP-D3/6-311G* are in close agreement with the reported E_b_ for H_2_ (−4.52 eV) [43,44], formation energy (E_f_) of pristine C_24_N_24_ (−7.40 eV), and E_b_ of Ti_6_@C_24_N_24_ (−8.14 eV), as well as the geometry values for C-C (1.55 Å) and C-N (1.34 Å) bond length [45].

The cohesive energy (per atom) of C_24_N_24_ is defined as:

E_coh_ = (1/48)(E_C24N24_ − 24 E_C_ − 24 E_N_) (1)

where the E_C24N24_, E_C_, and E_N_ are the calculated total energy of the pure nanocage, carbon, and nitrogen atom, respectively. The E_b_ of each doped TM in C_24_N_24_ for a homogeneous TM-doped C_24_N_24_ (TM_6_@C_24_N_24_) and with different metal ratios (x, y, z = 0–6) was calculated using Equations (2) and (3), respectively:

E_b(TM6@C24N24)_ = (1/6)(E_TM6@C24N24_ − 6 E_TM_ − E_C24N24_) (2)

E_b(TixMnyCuz@C24N24)_ = (1/6)(E_TixMnyCuz-C24N24_ − E_C24N24_ − xE_Ti_ − yE_Mn_ − zE_Cu_), (x, y, z = 0–6) (3)

where, in Equation (2), the E_TM6@C24N24_ and E_TM_ refers to the total energy of one type TM-doped C_24_N_24_ (Ti, Mn, or Cu) and the TM atom, respectively. In Equation (3), the E_TixMnyCuz@C24N24_ is the total energy of the doped C_24_N_24_ complex and xE_Ti_, yE_Mn_, and zE_Cu_ are defined as the total energy of Ti, Mn, and Cu metal atoms times the number of doped Ti atoms (x), Mn atoms (y), and Cu atoms (z) into the six porphyrin cavities of C_24_N_24_ nanocage, respectively.

The adsorption energy (E_ads_) of each gas moiety over the modified cage is defined as:

E_ads_ = E_ads@TixMnyCuz-C24N24_ − E_TixMnyCuz-C24N24_ − E_adsorbate_ (4)

where E_ads@TixMnyCuz-C24N24_, E_TixMnyCuz-C24N24_, and E_adsorbate_ are the total energy of the adsorbate on the complex, pure complex, and the adsorbate molecule, respectively. The x, y, z values represent the number of each TM atom doped into the porphyrin-like C_24_N_24_ cavities and are in the range of 0 to 6.

For each system, the zero-point energy (E_ZPE_) is calculated by summing vibrational frequencies over all (real) normal modes. Enthalpy and Gibbs energy changes are calculated at 298.15 K following the standard procedure outlined in the reference [46] To follow the nature of the adsorption process, the Wiberg bond indices (WBIs) were computed using NBO analysis. The WBIs are known as a better measure of bond strength relative to the overlap population, which is basis-set-dependent and often does not correlate well to bond strength. The WBIs are often similar in magnitude to the bond order expected from valence bond theory and have been used to propose trigger bonds in various energetic materials.

The WBI is a measure of the density between two atoms A and B. It determines by the sum of the off-diagonal square of the density matrix P (p#q) [47]:

(5) $${\mathrm{WBI}}_{\mathrm{AB}}=\sum_{P^{A}}\sum_{q^{B}}P^{2}{}_{\mathrm{pq}}$$

## 3. Results

### 3.1. Geometry of Pristine C_24_N_24_

**Figure 1:**
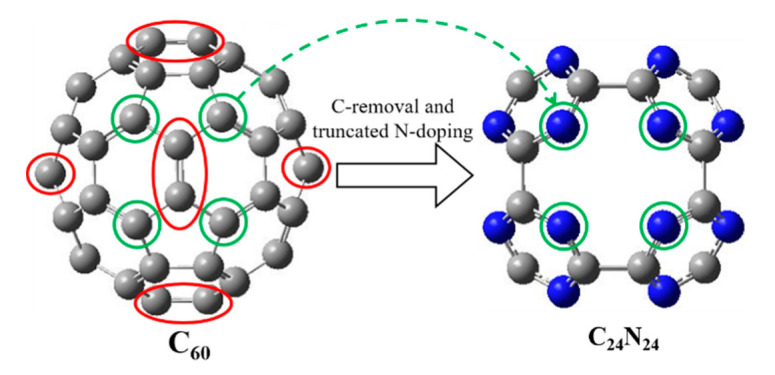
The optimized structure of porous C_24_N_24_ originated from fullerene. Color code: C, gray; N, blue.

The porous C_24_N_24_ is formed by first removing the 12 C atoms in C_60_ that connect two pentagons, thus creating six di-vacancies, as shown with a red circle in Figure 1, and subsequently substituting four undercoordinated C-atoms with N-atoms, thus creating six porphyrin-like N_4_ cavities and eight connected s-triazine rings (see Figure 1). The calculated C-C and C-N bond lengths are 1.55 Å and 1.34 Å, respectively. The calculated cohesive energy per atom in C_24_N_24_ is E_coh_ = −7.81 eV, which is close to that reported by Ghosh et al. (−7.40 eV) [45] but significantly higher than the value of Tang et al. (−5.78 eV) [38].

The HOMO-LUMO gap of C_24_N_24_ is calculated to be to be E_g_ = 2.82 eV, which is similar to the value reported by Ma et al. [39] and Song et al. [48] and is higher than the value reported in other investigations [40,45].

### 3.2. Metal Distribution Patterns

#### 3.2.1. Geometry and Electronic Properties of Bi-Metal Complexes

**Figure 2:**
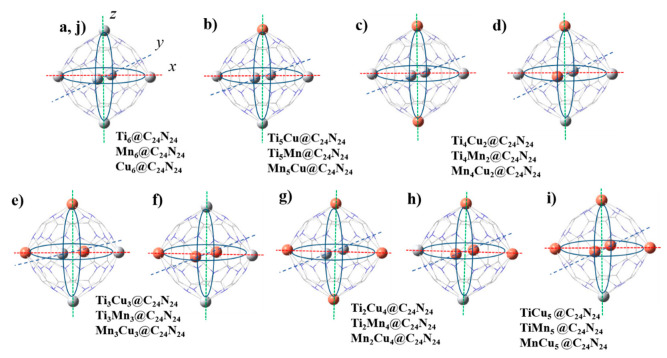
A schematic representative of possible distribution patterns of two dissimilar TMs (TM_1_:TM_2_) in N_4_-pyridinic cavities of the C_24_N_24_ nanocage: (**a**) 6:0, (**b**) 5:1, (**c**,**d**) 4:2, (**e**,**f**) 3:3, (**g**,**h**) 2:4, (**i**) 1:5, (**j**) 0:6.Color code: white, Ti; orange, Cu.

The agglomeration of catalytically active TMs into clusters is a major challenge. This can be prevented by the strong interaction between TMs and support. C_24_N_24_ fullerene possesses natural N_4_ rings that can host TM atoms (see Figure 1). We considered a combination of two metal atoms for the selected Ti, Mn, and Cu TMs with 3d^2^4s^2^, 3d^5^4s^2^, and 3d^10^4s^1^ valence electrons, respectively, i.e., TiCu, TiMn, and MnCu. Then, various distribution patterns of these dissimilar bi-metal atoms into the six porphyrin-like C_24_N_24_ cavities were studied. Due to the presence of six cavities, the TM ratios will vary between zero to six. Therefore, we have Ti_x_Cu_z_, Ti_x_Mn_y_, and Mn_y_Cu_z_ doped C_24_N_24_ fullerene (Ti_x_Cu_z_@C_24_N_24_, Ti_x_Mn_y_@C_24_N_24_, Mn_y_Cu_z_@C_24_N_24_) with x, y, z = 0–6. Figure 2a–j shows a schematic presentation of possible bi-metal distribution patterns in the N_4_ cavities of C_24_N_24_ cage. There are two main doping sites available in the C_24_N_24_ cavities: equatorial and axial. As can be seen in Figure 2, equatorial and axial positions are placed in x, y (red and black dashed lines), and *z* (green dashed line) axis directions, respectively. Two metal atoms in a complex can be distributed into the C_24_N_24_ cavities through seven different distribution patterns, as listed in Table 1.

#### 3.2.2. Binding Energy (E_b_)

**Figure 3:**
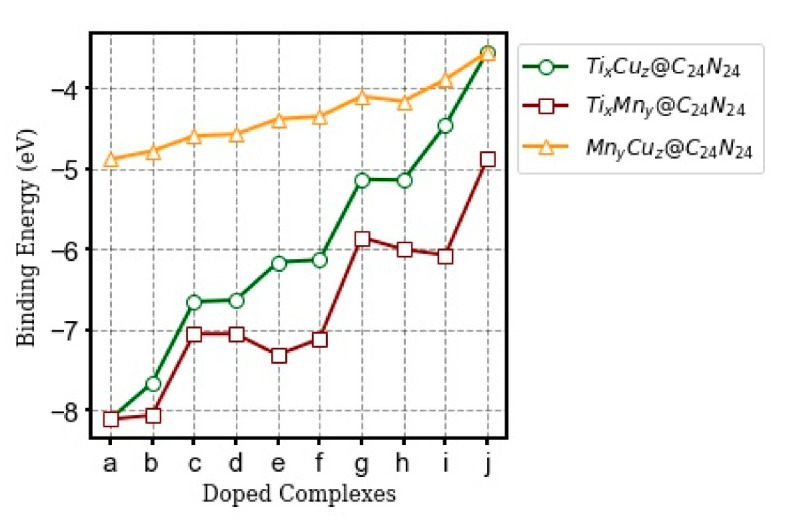
The calculated binding energy (E_b_) of Ti_x_Cu_z_@C_24_N_24_, Ti_x_Mn_y_@C_24_N_24_, and Mn_y_Cu_z_@C_24_N_24_ (x, y, z = 0–6). a–j refer to the bi-metal configurations with different metal ratios listed in Table 1. All values are in eV.

To assess the stability of the modified complexes, the binding energy of each configuration was calculated (see Tables S2–S4). The computed E_b_ for each complex changes in the range of −3.55 eV to −8.11 eV. The lowest and highest E_b_ values correspond to the homogeneous doping with Cu (Cu_6_@C_24_N_24_) and Ti (Ti_6_@C_24_N_24_), respectively. The calculated E_b_ for Ti_6_@C_24_N_24_ is in good agreement with previous studies (−8.61 eV and −8.14 eV) [39,45]. The binding energy of Ti_x_Cu_z_@C_24_N_24_, Ti_x_Mn_y_@C_24_N_24_, and Mn_y_Cu_z_@C_24_N_24_ (x, y, z = 0–6) configurations is plotted and compared in Figure 3.

The high E_b_ values indicate a strong chemisorption of two dissimilar TMs at six N_4_ cavities of C_24_N_24_, which inhibits the diffusion of TMs into the C_24_N_24_ fullerene, increasing the stability of nanocages by metal doping. Due to the higher binding energy of Ti_6_@C_24_N_24_ and Mn_6_@C_24_N_24_ than the cohesive energy of bulk Ti (4.85 eV/atom) and Mn (2.92 eV/atom) [49], TM aggregation is not expected, and these materials are likely to be stable enough to be used in catalytic processes. In contrast, Cu_6_@C_24_N_24_ shows an E_b_ of −3.55 eV, slightly higher than the cohesive energy of bulk Cu (3.49 eV/atom) [50], such that homogeneously Cu-doped C_24_N_24_ is much less stable against TM agglomeration.

One can see from Figure 3 and Tables S2–S4 that by increasing the ratio of Cu-to-Ti, Mn-to-Ti, and Cu-to-Mn atoms in Ti_x_Cu_z_@C_24_N_24_, Ti_x_Mn_y_@C_24_N_24_, and Mn_y_Cu_z_@C_24_N_24_, the E_b_ decreases (i.e., becomes less negative).

#### 3.2.3. NBO Charge Analysis

**Figure 4:**
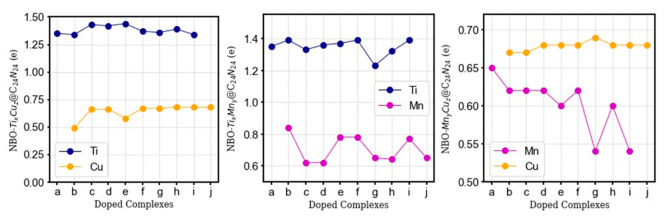
NBO charge analysis of individual Ti, Mn, and Cu atoms in Ti_x_Cu_z_@C_24_N_24_, Ti_x_Mn_y_@ C_24_N_24_, and Mn_y_Cu_z_@ C_24_N_24_. The a–j complexes refer to the metal distribution patterns presented in Figure 2. All values are in eV.

Figure 4 shows the NBO charge accumulation on individual TMs in different configurations (a–j) for each complex. The precise values are reported in Tables S2–S4. Ti and Cu atoms in Ti_x_Cu_z_@C_24_N_24_ and Mn_y_Cu_z_@C_24_N_24_ complexes have the highest charge transfer values from the metal atom to the nanocage, in the range of 1.34 to 1.44 |e| and 0.67 to 0.69 |e|, respectively. The high charge on the metal atoms corresponds to the high electron donation induced by the four surrounding N atoms in each porphyrin-like cavity where the d orbitals of Ti, Mn, and Cu atoms overlap with the neighboring nitrogen sp^2^ orbitals of the cavity to form a sp^2^d hybridization. The charge transfer leads to the elongation of the average C-C bond (≈1.55 Å) compared to that of pristine C_60_ (1.45 Å), indicating the activation of C_24_N_24_ fullerene upon bi-metal doping.

#### 3.2.4. Thermodynamic Properties and Energy Gap (E_g_)

**Figure 5:**
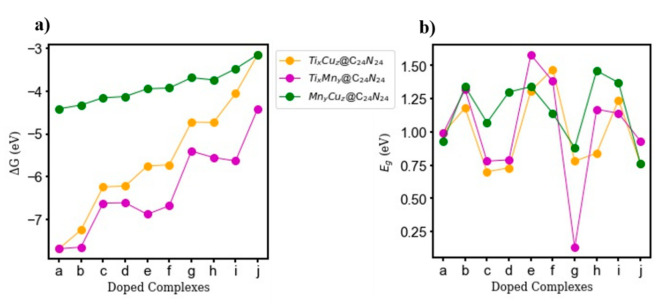
(**a**) The calculated changes in free energy (ΔG, at 298 K and 1 atm) (**b**) and energy gap for Ti_x_Cu_z_@C_24_N_24_, Ti_x_Mn_y_@C_24_N_24_, and Mn_y_Cu_z_@C_24_N_24_ (x, y, z = 0–6). a–j refer to the bi-metal configurations with different metal ratios listed in Table 1.

Figure 5 shows the changes in Gibbs free energy for each doped configuration, showing that bi-metallic doping is exothermic and exergonic at room temperature. The values are reported in Tables S2–S4.

Doping C_24_N_24_ with various Ti, Mn, or Cu distributions significantly narrows the HOMO-LUMO gap of the C_24_N_24_, leading to a noticeable energy gap (E_g_) reduction. The highest E_g_ reduction occurs in Ti_2_Mn_4_@C_24_N_24_, where the calculated E_g_ is reduced from 2.82 eV in pure C_24_N_24_ to 0.13 eV, making the complex act as a semi-conductor (see Figure 5b, complex g, purple line). The calculated HOMO−LUMO gap for doped configurations does not follow any particular order but in general is less than <1.58 eV, and thus all configurations can be classified as semiconductors. Since the electrical conductivity is exponentially related to E_g_, we expect that Ti_2_Mn_4_@C_24_N_24_ may show good electrical conductivity.

Besides the effects of doping on the geometric, thermodynamic, and electronic properties of the complexes as described above, we find that the location of the second introduced TM atom in axial or equatorial positions has little effect. Therefore, we expect that the complexes c and d, e and f, and g and h exhibit similar catalytic activities.

## 4. Catalytic Behavior of Bi-Metal Complexes toward Adsorption of Gas Species

### 4.1. Geometric Properties

To explore the effect of bi-metal-doping on the catalytic behavior of each complex, we investigated the individual adsorption of four gas molecules (CO_2_, NO_2_, H_2_, and N_2_) on each complex and their subgroup configurations. As shown in previous investigations, the existence of two dissimilar TM atoms in a catalyst induces various active sites [51,52,53,54]. Thus, it is important to first determine the available active sites in each substrate (see Figure S1). Considering each complex as a sphere, TM atoms are placed in x, y, and z directions. In homogeneous doping with one TM atom, one active site will be available (Figure S1a–j). By adding the second TM atom to the complex, it can be doped into one of the porphyrin vacancies along the x, y, or z direction, forming the complexes that are shown in Figure S1b. With two different TM atoms present in the complex, one would expect to have two different active sites available. However, for the adsorbent, there are not only two sites. We have labeled the TM atoms in Figure S1 (denoted as *i*, *j*, and *k*) to show different possible positions for the adsorbents to adsorb on the TM sites of each catalyst. It is clear that *i*, *k*/*j* sites are located on the axial (perpendicular to the plane of the ring)/equatorial (in the plane of the ring) axis of the catalyst, respectively. Depending on the TM ratio, the adsorbent can adsorb on either *i*, *j*, or *k* positions. One can see that for each configuration, two active sites are available except in configurations b and i, in which, depending on the adsorption position of the adsorbent in the equatorial (*j*) or axial (*i* and *k*) axis, three possible active sites are available. In addition, each adsorbent adopts mainly three adsorption modes on each active site of the substrates: parallel (side-on) or vertical (end-on) to the surface, and bridge positions.

**Figure 6:**
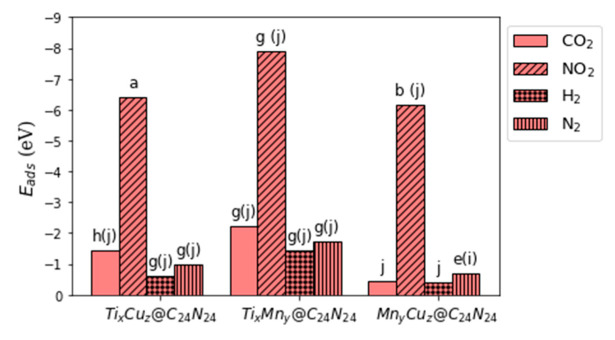
The more active bi-metal configurations of Ti_x_Cu_z_@C_24_N_24_, Ti_x_Mn_y_@C_24_N_24_, and Mn_y_Cu_z_@C_24_N_24_ (x, y, z = 0–6) toward CO_2_, NO_2_, H_2_, and N_2_ gas capture.

Our results reveal that CO_2_, H_2_, and N_2_ adsorb strongly on Ti_x_Cu_z_@C_24_N_24_ and Ti_x_Mn_y_@C_24_N_24_ and weakly on Mn_y_Cu_z_@C_24_N_24_, while NO_2_ adsorbs quite strongly with |E_ads_| > 6 eV on all three complexes (see Figure 6 and Table S5). The adsorption behavior of each set of configurations is discussed below.

### 4.2. Adsorption of CO_2_ and H_2_

**Figure 7:**
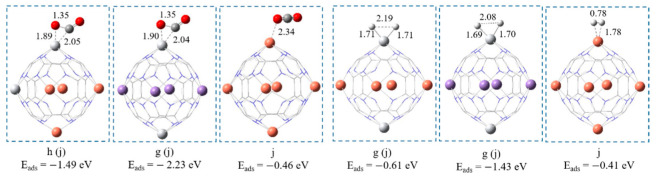
The optimized configurations of CO_2_ and H_2_ on bi-metal complexes. All bond distances are in Å. Color code: white, Ti; orange, Cu; purple, Mn; red, O; grey, C; blue, N.

Ti_2_Cu_4_@C_24_N_24_ and Ti_2_Mn_4_@C_24_N_24_ tend to chemisorb CO_2_ and H_2_ species with high adsorption energies (see Figure 7). The covalent nature of the adsorbed species is confirmed by the calculated WBIs. As a result, the structure of CO_2_ is drastically distorted upon its adsorption on these complexes. It is bent over the Ti atom binding via its C and O and forms a triangular ring above the nanocage. The O-C-O angle is bent to 131.12° in its adsorbed form and the C = O bond length is elongated to 1.35 Å, which we attribute to the significant charge transfer of 0.49 |e| from the complex to the 2π* orbitals of the CO_2_ molecule. The obtained adsorption energies for CO_2_ are lower than those reported on B_80_ fullerene (−3.49 eV) [55] and higher than those of N-S dual doped graphene (−0.25 eV) [56] and Ti-doped C_2_N (E_ads_ = −0.95 eV) [57]. The WBIs of both Ti-C and Ti-O bonds in Ti_2_Cu_4_@C_24_N_24_ and Ti_2_Mn_4_@C_24_N_24_ are 0.84, confirming the covalent bond between TM and CO_2_ atoms and therefore its chemisorption over these substrates. H_2_ adsorbs dissociatively in a barrier-less reaction, forming two covalent Ti-H bonds above the Ti atom with H-H bond length of >2.0 Å (see Figure 7). The NBO charge analysis (see Table S5) along with the calculated WBIs for both Ti-H bond lengths (0.89) confirms the covalent binding between Ti and H atoms and consequently the H_2_ chemisorption. Interestingly, we find that the adsorption energy of one hydrogen molecule on these complexes is higher than the adsorption of six H_2_ molecules on Ti_6_@C_24_N_24_ (E_ads_ = −0.48 eV) [39] [39], Fe-B_38_ (E_ads_ = −0.42 eV), Co-B_38_ (E_ads_ = −0.72 eV), Ni-B_38_ (E_ads_ = −0.89 eV) [58], and 2D carbon allotrope Ψ graphene (E_ads_ = −0.34 eV) [59].

Due to the lower activity of the Cu_6_@C_24_N_24_ complex, CO_2_ and H_2_ physisorb on this structure in their gas-phase form. The calculated E_ads_ of H_2_ on Cu_6_@C_24_N_24_ is lower than that reported on Ti_2_C- and Ti_2_CN-Mxenes (E_ads_ in the range of −0.99 to −1.4 eV) [60]. A negligible charge transfer from CO_2_ (H_2_) to Cu_6_@C_24_N_24_, the large Cu-C (Cu-H) bond length, and the low WBIs value of 0.12 (0.2) confirm physisorption of these CO_2_ (H_2_) species on Cu_6_@C_24_N_24_.

### 4.3. Adsorption of N_2_ and NO_2_

**Figure 8:**
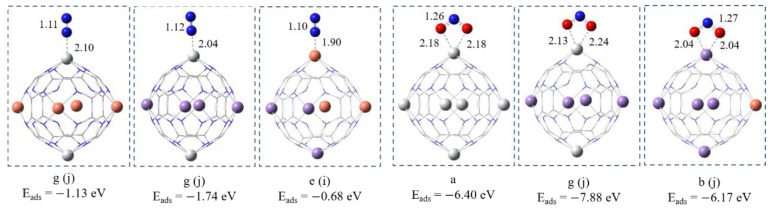
The optimized configurations of N_2_ and N_2_O on bi-metal complexes. All bond distances are in Å. Color code: white, Ti; orange, Cu; purple, Mn; red, O; grey, C; blue, N.

Two different orientations were considered for N_2_ adsorption on each structure: side-on or end-on. The ideal catalyst would provide strong binding sites for the N_2_ molecule and thus weaken the N−N bond. Our results indicate that the ideal orientation for N_2_ adsorption on Ti_2_Cu_4_@C_24_N_24_ and Ti_2_Mn_4_@C_24_N_24_ and Mn_3_Cu_3_@C_24_N_24_ is the end-on (see Figure 8). Upon N_2_ adsorption, the N-N bond length increases from 1.09 Å in the gas phase to 1.11 and 1.12 Å in its adsorbed form on Ti_2_Cu_4_@C_24_N_24_ and Ti_2_Mn_4_@C_24_N_24_ complexes, respectively. These values are in between the double and triple bond lengths, indicating the activation of N_2_ upon adsorption on Ti sites. The empty d orbitals of the Ti atom can accept the lone-pair electrons of N_2_. In turn, the Ti’s capacity to donate electrons to the antibonding π* orbital of N_2_ is also significant for N_2_ binding to Ti. Therefore, this electron acceptance/donation process between the nanocage and N_2_ plays an important role in N_2_ activation (see Table S5). Ti_2_Mn_4_@C_24_N_24_ has a greater tendency for N_2_ activation with higher adsorption energy than that reported on Co-doped graphitic carbon nitride (−1.63 eV) [61] and Fe doped phosphorene (−0.81 eV) [62].

Although the Mn_3_Cu_3_@C_24_N_24_ complex adsorbs N_2_ molecule with lower E_ads_, due to its lower catalytic activity toward gas adsorption, this value is still higher than that on Mn-Fe bi-metal atoms anchored pyridinic nitrogen-doped graphene (E_ads_ = −0.53 eV) [63]. The calculated WBIs (≈0.0001) and charge transfer confirm the N_2_ physisorption on Mn_3_Cu_3_@C_24_N_24_.

Regardless of all possible bi-metal distributions into the porphyrin C_24_N_24_ cavities, the three Ti_x_Cu_z_@C_24_N_24_, Ti_z_Mn_y_@C_24_N_24_, and Mn_y_Cu_z_@C_24_N_24_ (x, z, y = 0–6) complexes with all the metal ratios exhibit an outstanding activity toward NO_2_ adsorption and activation. However, the more stable configurations are shown in Figure 8. One can see that the NO_2_ molecule binds via two O atoms with the Ti atom in Ti_6_@C_24_N_24_ and Ti_2_Mn_4_@C_24_N_24_ and with the Mn atom in the Mn_5_Cu@C_24_N_24_ complex, respectively. Owing to the great NBO charge transfer from the nanocage to the NO_2_ molecule (0.46 |e|) reported in Table S5, the N-O bond length increases compared to that of the gas phase (1.19 Å).

### 4.4. Electronic and Thermodynamic Properties

To see if the adsorption of CO_2_, NO_2_, H_2_, and N_2_ gas species on the selected twelve more energetically stable complexes affects their electronic properties, we also investigated the LUMO-HOMO energy gap of each structure and compared our results with those of pristine bi-metal doped nanocages reported in Figure 5. The obtained E_g_ values for adsorption structures are listed in Table S5, showing that E_g_ indeed increases upon gas adsorption. This confirms the tunable electronic properties of C_24_N_24_ nanocage induced by hosting six dissimilar bi-metals doping into its N_4_ cavities with various distribution patterns. All adsorption reactions are exothermic and exergonic, except for H_2_ adsorbed on the Cu_6_@C_24_N_24_ complex, which is slightly endergonic (see Table S5).

### 4.5. Lifetime of the Adsorbed Gas Species on Bi-Metal Complexes

**Figure 9:**
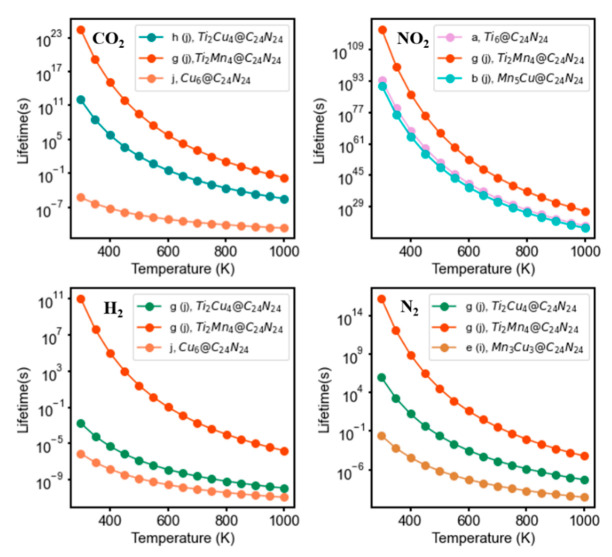
The calculated lifetime of the adsorbed gas species on Ti_x_Cu_z_@C_24_N_24_, Ti_x_Mn_y_@C_24_N_24_, and Mn_y_Cu_z_@C_24_N_24_.

The retention time of a molecule on a surface can be calculated with the Frenkel equation [64]:

(6) τ=τ0eQRT

where $\tau_{0}$ is 10^−12^ to 10^−13^ s and *Q* is the adsorption energy. We calculated the lifetime of each adsorbent over each configuration from which the energetically more favorable structures were chosen and plotted versus temperature. Figure 9 shows the computed lifetime vs. temperature for adsorbed CO_2_, NO_2_, H_2_, and N_2_ on the twelve nanocages discussed above. As can be seen in Figure 9, the lifetime of gas species on Ti_x_Cu_z_@C_24_N_24_ and Ti_x_Mn_y_@C_24_N_24_ at 400 K is higher than that on Mn_y_Cu_z_@C_24_N_24_ (x, y, z = 0–6), indicating that Ti_x_Cu_z_@C_24_N_24_ and Ti_x_Mn_y_@C_24_N_24_ are likely to be more efficient for gas capture. Obviously, increasing the temperature reduces the lifetime leading. However, Ti_x_Cu_z_@C_24_N_24_, Ti_x_Mn_y_@C_24_N_24_, and Mn_y_Cu_z_@C_24_N_24_ catalysts capture NO_2_ so actively that it will not desorb from the catalyst even at high temperatures. Therefore, we can conclude that, except for NO_2_, which effectively adsorbs and is retained on the catalysts, the gas species adsorb and remain on the Ti active site of Ti_x_Cu_z_@C_24_N_24_ and Ti_x_Mn_y_@C_24_N_24_ fullerene at room temperature, whereas they are not retained on Mn_y_Cu_z_@C_24_N_24_ sufficiently for gas capture.

## 5. Conclusions

In this work, the effect of dissimilar bi-metal doping into the six porphyrin-like cavities of a C_24_N_24_ nanocage on the catalytic activity and adsorption characteristic of a number of greenhouse gases are investigated by means of DFT calculations. The binding energy and bulk cohesive energy calculations reveal that the selected TM atoms are stably trapped in the C_24_N_24_ cavities, especially Ti/Cu atoms in Ti_x_Cu_z_@C_24_N_24_ and Ti/Mn atoms in Ti_x_Mn_y_@C_24_N_24_ (x, y, z = 0–6) complexes, suggesting the durability of the catalysts. Studying the adsorption behavior of these catalysts toward H_2_, CO_2_, NO_2_, and N_2_ sensing show that the Ti_2_Mn_4_@C_24_N_24_ is more active for adsorption of all species. Furthermore, the lifetime of each gas species on Ti_x_Cu_z_@C_24_N_24_ and Ti_x_Mn_y_@C_24_N_24_ at 400 K is higher than that on Mn_y_Cu_z_@C_24_N_24_ (x, y, z = 0–6)_,_ indicating that Ti_x_Mn_y_@C_24_N_24_ and Ti_x_Cu_z_@C_24_N_24_ are likely to be more efficient for gas capture. Overall, this work systematically provides the unique fundamental understanding of catalytic properties of two dissimilar bi-metal atom catalysts that could open a way for the future design and development of novel few-atom catalysts.

## Figures and Tables

**Table 1:** Distribution patterns of bi-metal atoms in C_24_N_24_ cavities.

Complex/Metal-Ratio	6:0	5:1	4:2	3:3	2:4	1:5	0:6
	a	b	c, d	e, f	g, h	i	j
Ti_x_Cu_z_@C_24_N_24_	Ti_6_	Ti_5_Cu	Ti_4_Cu_2_	Ti_3_Cu_3_	Ti_2_Cu_4_	TiCu_5_	Cu_6_
Ti_x_Mn_y_@C_24_N_24_	Ti_6_	Ti_5_Mn	Ti_4_Mn_2_	Ti_3_Mn_3_	Ti_2_Mn_4_	TiMn_5_	Mn_6_
Mn_y_Cu_z_@C_24_N_24_	Mn_6_	Mn_5_Cu	Mn_4_Cu_2_	Mn_3_Cu_3_	Mn_2_Cu_4_	MnCu_5_	Cu_6_

## Data Availability

Not applicable.

## Supplementary Materials

The following are available online at https://www.mdpi.com/article/10.3390/nano11071794/s1, Figure S1: A schematic representative of available active sites (shown with red stars) on various BM@C_24_N_24_ configurations.Color code: white, Ti; orange, Cu., Table S1: The calculated cohesive energy (Ecoh) per atom of C_24_N_24_, binding energy (E_b_) of hydrogen molecule and Ti_6_-doped C_24_N_24_ along the energy gap of pristine C_24_N_24_ using different functional and basis sets, Table S2: The calculated binding energy (E_b_), change of enthalpy (ΔH_298_), change of Gibbs free energy (ΔG_298_), energy gap (E_g_), and the average NBO charge on Ti_x_Cu_z_@C_24_N_24_. All the values are in eV, Table S3: The calculated binding energy (E_b_), change of enthalpy (ΔH_298_), change of Gibbs free energy (ΔG_298_), energy gap (E_g_), and the average NBO charge on Ti_x_Mn_y_@C_24_N_24_. All the values are in eV, Table S4: The calculated binding energy (E_b_), change of enthalpy (ΔH_298_), change of Gibbs free energy (ΔG_298_), energy gap (E_g_), and the average NBO charge on Mn_y_Cu_z_@C_24_N_24_. All the values are in eV, Table S5: The calculated total adsorption energy (E_ads_), energy gap (E_g_), changes of enthalpy(ΔH_298_), changes of free energy(ΔG_298_), and NBO charge analysis for the energetically more stable adsorption configurations.
