# Supplementary material for: Linking Bi-Metal Distribution Patterns in Porous Carbon Nitride Fullerene to Its Catalytic Activity toward Gas Adsorption
Source: Nanomaterials (Basel). 2021 Jul 9;11(7):1794. doi: 10.3390/nano11071794 (PMC8308207; doi:10.3390/nano11071794)
Supplement: Supplementary file 1 [file nanomaterials-11-01794-s001.zip › nanomaterials-1257511-supplementary.pdf]

Article

Linking Bi-Metal Distribution Patterns in Porous Carbon Nitride Fullerene to its Catalytic Activity toward Gas Adsorption

Parisa Nematollahi * and Erik C. Neyts

Research Group Plasmant, NANO Lab Center of Excellence, Department of Chemistry, University of Antwerp, 2610 Antwerp, Belgium; Erik.Neyts@uantwerpen.be
* Correspondence: parisa.nematollahi@uantwerpen.be; Tel.:+32-32-652-346

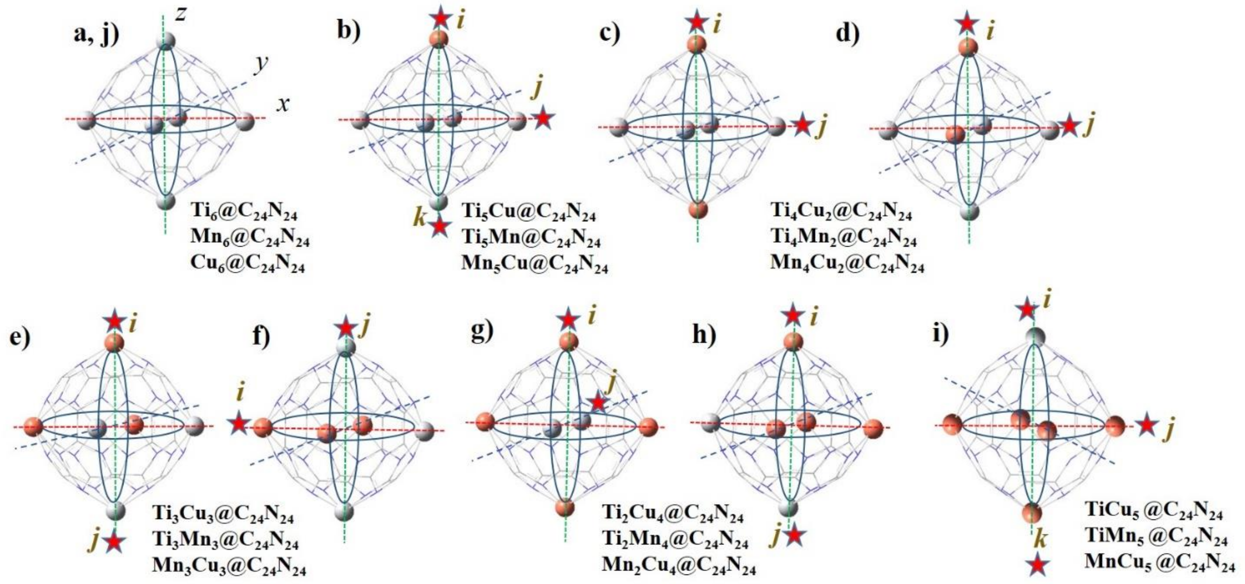

Figure S1. A schematic representative of available active sites (shown with red stars) on various $BM@C_{24}N_{24}$ configurations. Color code: white, Ti; orange,Cu.

The selection of a suitable functional and basis-set

Table S1 shows 14 different functional/basis sets that are often used in the literature. The chosen functionals are selected from various functional classes including double hybrid (DSDPBEP86 as a dispersion-corrected double hybrid functional with Grimme's D3BJ dispersion, PBE0DH, and PW6B95D3 [1]), Becke Three-Parameter Hybrid Functionals (B3LYP, P3LYPD3), functionals including dispersion with long-range correction (wB97XD [2], from Head-Gordon and coworkers, which includes empirical dispersion), a functional from the Truhlar Group (PW6B95D3 [1]), hybrid functionals of Truhlar and Zhao [3] (M06, M062X , M062XD3) as global-hybrid meta-GGA functionals, and finally B97D3 [4].

Table S1. The calculated formation energy (E_f) per atom of $C_{24}N_{24}$, binding energy (E_b) of hydrogen molecule and Ti_6-doped $C_{24}N_{24}$ along the energy gap of pristine $C_{24}N_{24}$ using different functional and basis sets.

Number	1	2	3	4	5	6	7	8	9	10	11	12	13	14
Functional	M062x	DSDPBEP86	PW6B95D3	B2PLYPD3	B3LYP	M06	PBE0DH	B3LYPD3	M062x	B97D3	M062X	M062x	M062XD3	wB97XD
Basis-set	6-311G*								6-311G (2df, 2pd)		6-31G*	6-311++G(2df,2pd)		
E_f ($C_{24}N_{24}$)	-7.87	-7.05	-4.06	-7.21	-7.76	-3.90	-4.04	-7.81	-3.99	-4.05	-7.99	-3.95	-3.96	-4.00
E_b (H_2)	-4.31	-4.04	-4.33	-4.20	-4.42	-4.33	-4.10	-4.42	-4.42	-4.60	-4.36	-4.42	-4.42	-4.38
E_g	5.52	7.01	3.45	5.69	2.84	3.36	5.44	2.82	5.53	1.09	5.50	5.47	5.48	6.73
E_b (Ti_6-$C_{24}N_{24}$[a])	-	-	-8.22	-	-7.99	-	-	-8.14	-	-	-	-	-	-

[a] The basis set LanL2TZ is considered for Ti atoms.

Table S2. The calculated binding energy (E_b), change of enthalpy (ΔH_{298}), change of Gibbs free energy (ΔG_{298}), energy gap (E_g), and the average NBO charge on $Ti_xCu_z@C_{24}N_{24}$. All the values are in eV.

$Ti_xCu_z@C_{24}N_{24}$	E_b	ΔH_{298}	ΔG_{298}	E_g	q_{Ti}	q_{Cu}
$Ti_6@C_{24}N_{24}$(a)*	-8.11	-8.15	-7.69	0.99	1.35	-
$Ti_5Cu@C_{24}N_{24}$(b)	-7.66	-7.69	-7.25	1.18	1.34	0.49
$Ti_4Cu_2@C_{24}N_{24}$ (c)	-6.65	-6.69	-6.24	0.70	1.43	0.66
$Ti_4Cu_2@C_{24}N_{24}$ (d)	-6.63	-6.66	-6.22	0.73	1.42	0.66
$Ti_3Cu_3@C_{24}N_{24}$ (e)	-6.16	-6.18	-5.75	1.31	1.44	0.58
$Ti_3Cu_3@C_{24}N_{24}$ (f)	-6.13	-6.15	-5.72	1.47	1.37	0.67
$Ti_2Cu_4@C_{24}N_{24}$ (g)	-5.13	-5.16	-4.72	0.78	1.36	0.67
$Ti_2Cu_4@C_{24}N_{24}$ (h)	-5.14	-5.16	-4.73	0.84	1.39	0.68
$TiCu_5@C_{24}N_{24}$ (i)	-4.46	-4.48	-4.05	1.24	1.34	0.68
$Cu_6@C_{24}N_{24}$ (a)	-3.55	-3.57	-3.14	0.76	-	0.68

*the letters in parentheses refers to the schematic representation of bimetals distribution into the $C_{24}N_{24}$ cavities, Figure 2

Table S3. The calculated binding energy (E_b), change of enthalpy (ΔH_{298}), change of Gibbs free energy (ΔG_{298}), energy gap (E_g), and the average NBO charge on $Ti_xMn_y@C_{24}N_{24}$. All the values are in eV.

$Ti_xMn_y@C_{24}N_{24}$	E_b	ΔH_{298}	ΔG_{298}	E_g	q_{Ti}	q_{Mn}
$Ti_6@C_{24}N_{24}$(a)*	-8.11	-8.15	-7.69	0.99	1.35	-
$Ti_5Mn@C_{24}N_{24}$(b)	-8.06	-8.10	-7.65	1.32	1.39	0.84
$Ti_4Mn_2@C_{24}N_{24}$ (c)	-7.05	-7.10	-6.62	0.78	1.33	0.62
$Ti_4Mn_2@C_{24}N_{24}$ (d)	-7.05	-7.09	-6.61	0.79	1.36	0.62
$Ti_3Mn_3@C_{24}N_{24}$ (e)	-7.31	-7.35	-6.88	1.58	1.37	0.78
$Ti_3Mn_3@C_{24}N_{24}$ (f)	-7.11	-7.15	-6.68	1.38	1.39	0.78
$Ti_2Mn_4@C_{24}N_{24}$ (g)	-5.85	-5.90	-5.40	0.13	1.23	0.65
$Ti_2Mn_4@C_{24}N_{24}$ (h)	-6.00	-6.04	-5.55	1.17	1.32	0.64
$TiMn_5@C_{24}N_{24}$ (i)	-6.07	-6.11	-5.63	1.14	1.39	0.77
$Mn_6@C_{24}N_{24}$ (a)	-4.88	-4.93	-4.41	0.93	-	0.65

Table S4. The calculated binding energy (E_b), change of enthalpy (ΔH_{298}), change of Gibbs free energy (ΔG_{298}), energy gap (E_g), and the average NBO charge on $Mn_yCu_z@C_{24}N_{24}$. All the values are in eV.

$Mn_yCu_z@C_{24}N_{24}$	E_b	ΔH_{298}	ΔG_{298}	E_g	q_{Cu}	q_{Mn}
$Mn_6@C_{24}N_{24}$ (a)	-4.88	-4.93	-4.41	0.93	-	0.65
$Mn_5Cu@C_{24}N_{24}$ (b)	-4.78	-4.82	-4.32	1.34	0.67	0.62
Mn_4 $Cu_2@C_{24}N_{24}$ (c)	-4.59	-4.63	-4.15	1.07	0.67	0.62
$Mn_4Cu_2@C_{24}N_{24}$ (d)	-4.57	-4.61	-4.12	1.30	0.68	0.62
Mn_3 $Cu_3@C_{24}N_{24}$ (e)	-4.38	-4.41	-3.94	1.34	0.68	0.60
$Mn_3Cu_3@C_{24}N_{24}$ (f)	-4.35	-4.39	-3.92	1.14	0.68	0.62
$Mn_2Cu_4@C_{24}N_{24}$ (g)	-4.10	-4.14	-3.68	0.88	0.69	0.54
Mn_2 $Cu_4@C_{24}N_{24}$ (h)	-4.16	-4.19	-3.73	1.46	0.68	0.60
$MnCu_5@C_{24}N_{24}$(i)	-3.89	-3.92	-3.47	1.37	0.68	0.54
$Cu_6@C_{24}N_{24}$(j)*	-3.55	-3.57	-3.14	0.76	0.68	-

Table S5. The calculated total adsorption energy (E_{ads}), energy gap (E_g), changes of enthalpy(ΔH_{298}), changes of free energy(ΔG_{298}), and NBO charge analysis for the energetically more stable adsorption configurations.

Complex		Adsorbate	E_{ads}(eV)	E_{ads}^*(eV)	E_g(eV)	ΔH_{298}(eV)	ΔG_{298}(eV)	q_C(e)
$Ti_2Cu_4@C_{24}N_{24}$	h(j)	CO_2	-1.49	-1.42	1.20	-1.43	-0.97	-0.49
$Ti_2Mn_4@C_{24}N_{24}$	g(j)		-2.23	-2.23	1.25	-2.24	-1.78	-0.49
$Cu_6@C_{24}N_{24}$	j		-0.46	-0.31	0.90	-0.27	-0.01	0.06
$Ti_6@C_{24}N_{24}$	a	NO_2	-6.40	-5.93	1.15	-5.91	-5.50	-0.46
$Ti_2Mn_4@C_{24}N_{24}$	g(j)		-7.88	-7.75	1.16	-7.72	-7.32	-0.45
$Mn_5Cu@C_{24}N_{24}$	b(j)		-6.17	-5.65	1.55	-5.63	-5.18	-0.16
$Ti_2Cu_4@C_{24}N_{24}$	g(j)	H_2	-0.61	-0.35	1.01	-0.38	-0.07	-0.25
$Ti_2Mn_4@C_{24}N_{24}$	g(j)		-1.43	-1.37	1.29	-1.43	-1.08	-0.22
$Cu_6@C_{24}N_{24}$	j		-0.41	-0.24	0.91	-0.27	0.02	0.08
$Ti_2Cu_4@C_{24}N_{24}$	g(j)	N_2	-1.00	-0.74	1.04	-0.73	-0.33	-0.20
$Ti_2Mn_4@C_{24}N_{24}$	g(j)		-1.74	-1.69	0.82	-1.72	-1.27	-0.26
$Mn_3Cu_3@C_{24}N_{24}$	e(i)		-0.68	-0.61	1.34	-0.62	-0.25	0.04

*The calculated adsorption energy with the consideration of the zero-point energy (ZPE) correction

[1] Y. Zhao, D.G. Truhlar, Design of Density Functionals That Are Broadly Accurate for Thermochemistry, Thermochemical Kinetics, and Nonbonded Interactions, *J. Phys. Chem. A.* **2005**. *109*, 5656–5667.
[2] J.-D. Chai, M. Head-Gordon, Long-range corrected hybrid density functionals with damped atom–atom dispersion corrections, *Phys. Chem. Chem. Phys.* **2008**. *10,* 6615–6620.
[3] Y. Zhao, D.G. Truhlar, The M06 suite of density functionals for main group thermochemistry, thermochemical kinetics, noncovalent interactions, excited states, and transition elements: two new functionals and systematic testing of four M06-class functionals and 12 other functionals, *Theor. Chem. Acc.* **2008,** *120*, 215-241.
[4] A.D. Becke, Density-functional thermochemistry. V. Systematic optimization of exchange-correlation functionals, *J. Chem. Phys.* **1997,** *107,* 8554–8560.
